# Results of the ACCOuNT Trial: A Multi‐Institutional Prospective Pharmacogenomics Implementation Trial for African American Inpatients

**DOI:** 10.1002/cpt.70024

**Published:** 2025-08-22

**Authors:** Zhong Huang, Kevin J. O’Leary, Edith A. Nutescu, Matthew Jack, Thomas Chen, Gregory W. Ruhnke, James C. Lee, David George, Larry K. House, Randall Knoebel, Luke V. Rasmussen, Seth Hartman, Anish Choksi, Kiang‐Teck J. Yeo, Minoli A. Perera, Mark J. Ratain, David O. Meltzer, Peter H. O’Donnell

**Affiliations:** ^1^ Pritzker School of Medicine, The University of Chicago Chicago Illinois USA; ^2^ Division of Hospital Medicine, Department of Medicine Northwestern University Chicago Illinois USA; ^3^ Department of Pharmacy Practice University of Illinois Chicago Chicago Illinois USA; ^4^ Center for Personalized Therapeutics The University of Chicago Chicago Illinois USA; ^5^ Section of Hospital Medicine, Department of Medicine The University of Chicago Chicago Illinois USA; ^6^ Department of Pathology The University of Chicago Chicago Illinois USA; ^7^ Section of Hematology/Oncology, Department of Medicine The University of Chicago Chicago Illinois USA; ^8^ Department of Pharmacy The University of Chicago Chicago Illinois USA; ^9^ Division of Biostatistics and Informatics, Department of Preventive Medicine Northwestern University Feinberg School of Medicine Chicago Illinois USA; ^10^ Department of Pharmacology Northwestern University Chicago Illinois USA

## Abstract

Pharmacogenomics implementation efforts have increased over the last decade, but no published prospective pharmacogenomics trials have formally evaluated utility in underrepresented populations. We present final results from the ACCOuNT trial, a multi‐institutional prospective study [NCT03225820] in which African American inpatients were genotyped and pharmacogenomic results made available via an integrated, scalable pharmacogenomics clinical decision support (PGx CDS) system to guide prescribing. We hypothesized that PGx CDS utilization would improve genomically concordant prescribing during inpatient admissions. We recruited 531 patients, of which 187 were readmitted (60.4% female, median age = 55 [range: 19–88], median readmissions/patient = 2 [range: 1–17]), resulting in 518 evaluable admissions during which the effect of available pharmacogenomic results was measured. In 50% of admissions, care teams utilized PGx CDS. 12% of inpatient prescriptions had pharmacogenomic annotations, with only 3.6% pharmacogenomically associated with increased caution (“discordant”); no PGx high‐risk medications were prescribed. PGx CDS use by provider teams did not decrease genomically discordant prescribing (3.9% vs. 3.3% without PGx CDS use, *P* = 0.53). Nevertheless, inpatient care teams repeatedly consulted PGx CDS ostensibly to affirm genomically concordant prescriptions, as 48% of all admissions and 91% of discharges included pharmacogenomically annotated medications, mostly genomically concordant. Notably, timing of PGx CDS utilization correlated tightly with prescribing timing (correlation coefficient = 0.84, *P* < 0.05). In the first results of a multi‐institutional, prospective pharmacogenomics trial focused on underrepresented patients, we found the relevance of currently actionable pharmacogenomics information for guiding inpatient prescribing to be modest; but observed frequent use of concordant pharmacogenomics to affirm prescribing.

**Study Highlights**

**WHAT IS THE CURRENT KNOWLEDGE ON THIS TOPIC?**

Pharmacogenomic (PGx) implementation efforts have proliferated over the last decade, but there are currently no published multi‐institutional PGx implementation studies focused on underrepresented populations.

**WHAT QUESTION DID THIS STUDY ADDRESS?**

We implemented a broad, preemptive PGx clinical decision support (CDS) system across three institutions as part of a large, prospective, pragmatic clinical trial focusing on the utility of providing PGx information during the care of self‐identified African American inpatients. We hypothesized that PGx CDS utilization would improve genomically concordant prescribing during admissions.

**HOW DOES THIS STUDY ADD TO OUR KNOWLEDGE?**

The relevance of currently actionable PGx information for guiding inpatient medicine prescribing among African Americans was modest, with no contraindicated (high risk) medications and a low percentage of potentially incongruent (increased caution) prescriptions. While this led to the fact that PGx CDS did not measurably impact genomically discordant prescribing, we nonetheless found that inpatient care teams frequently utilized concordant PGx CDS as prescribing reassurance.

**HOW MIGHT THIS CHANGE CLINICAL PHARMACOLOGY OR TRANSLATIONAL SCIENCE?**

In the first results of a multi‐institutional, prospective PGx implementation trial focusing on the care of underrepresented patients, we establish novel evidence for the use of PGx information to affirm inpatient prescribing decisions.

Pharmacogenomics (PGx) implementation resources have grown over the last decade, often leveraging clinical decision support (CDS) tools to deliver prescribing guidance within the electronic health record (EHR).^1^ However, few implementation efforts have been focused on the benefit of underrepresented minority groups.^2^, ^3^, ^4^ The majority of PGx implementation studies report < 20% representation by individuals of African ancestry, for example, with most including far lower percentages than that.^5^, ^6^, ^7^, ^8^, ^9^, ^10^, ^11^, ^12^, ^13^, ^14^, ^15^, ^16^, ^17^, ^18^ Among studies that did focus on African Americans, small sample sizes (often below 100 participants) limit the generalizability of the findings.^2^, ^3^, ^4^ These implementation gaps represent a potential disparity in opportunities to benefit from genomic technologies for medication use.^19^, ^20^ It also means that the utility of PGx information remains poorly understood within underrepresented populations.^19^, ^20^, ^21^ In response, in 2016, the United States National Institute on Minority Health and Health Disparities (NIMHD) funded the African American Cardiovascular Pharmacogenetic Consortium (ACCOuNT), which included both PGx “discovery” and “implementation” (translational) projects.^22^, ^23^

Beyond its focus on underrepresented populations, the translational project within ACCOuNT also had the goal of solving multi‐institutional, scalable delivery of PGx results at the point of care, a known challenge within translational PGx.^12^, ^13^, ^14^, ^15^, ^16^, ^24^, ^25^, ^26^, ^27^ Implementation descriptions from prior efforts have reported on necessary site variations in clinical workflows, EHR build specifications, and institutional informatics capacities that have resulted in significant differences in PGx information delivery between sites.^12^, ^13^, ^16^, ^17^, ^24^, ^25^, ^27^, ^28^ Even the recently completed European PREPARE trial—a groundbreaking effort for its scope and impact—encountered similar challenges, with sites differing in PGx CDS deployment settings (inpatient and/or outpatient), method (automatic EHR alerts, paper‐based PGx reports, digital PGx reports, and/or a plastic “Safety‐Code card”), and data structures (structured laboratory results and/or utilization of laboratory information management systems).^28^, ^29^ Almost all prior efforts have utilized clinical workflow adaptations—likely out of necessity because of informatics infrastructure variations across medical centers. These variations may limit each project’s ability to draw generalized conclusions about the clinical impact of PGx CDS, which is necessary to inform the development of standardized PGx CDS and adoption into routine care.^30^

To address these unmet needs, we launched the first ever multi‐institutional, prospective, pragmatic PGx implementation trial specifically evaluating African American patients, utilizing a scalable EHR‐integrated PGx CDS that displayed prescribing recommendations through a dynamic interface.^31^ Our hypothesis was that PGx CDS use would improve genomically concordant prescribing during inpatient hospitalizations for self‐identified African Americans. We herein report the final results of the ACCOuNT Trial.

## MATERIALS AND METHODS

### Study setting

The ACCOuNT Trial (clinicaltrials.gov NCT03225820) implemented broad preemptive PGx result delivery at three academic hospitals: The University of Chicago Medical Center (UCM), Northwestern Memorial Hospital (NW), and the University of Illinois Hospital & Health Sciences System (UIC). The Institutional Review Board of all three institutions approved this study, with UCM as the coordinating site.^22^

### Patient and provider enrollment and participation

Study providers were recruited from the hospital medicine and internal medicine services at each institution. Eligible providers included physicians, pharmacists, physician assistants, and advanced practice nurses who were likely to care for enrolled patients during the course of the study. Prior to participation, providers were consented to the study, required to complete a baseline questionnaire assessing knowledge and attitudes toward PGx, and were required to complete a brief (< 10 minutes) in‐person or online video‐based training on the use of the PGx CDS system. The study team also presented this study at multiple hospital medicine faculty meetings during the course of the study period to provide study background, facilitate engagement, and respond to questions or feedback. After enrollment, providers were regularly surveyed on their PGx CDS experience throughout the trial.

Per NIMHD parameters, self‐identified adult African American inpatients being treated at any of the three hospitals within the pre‐specified medicine services were eligible to be approached for participation (during their inpatient hospitalization). Subjects were enrolled using a process of informed consent from November 3, 2016, to March 25, 2021. We excluded patients who had undergone or were being considered for liver or kidney transplantation, those with active or past leukemia, and patients who were unable to provide informed consent.

### Patient genotyping procedures

Patients provided a peripheral blood sample at enrollment. Each sample was genotyped using a custom PGx panel covering 45 genes linked to 65 medications.^32^ Genotyping was conducted by the Advanced Technology PGx Clinical Laboratory at UCM, which is certified by the Clinical Laboratory Improvement Amendments and accredited by the College of American Pathologists.^32^ Patient data was securely stored on a Health Insurance Portability and Accountability Act‐compliant server at UCM.

### PGx CDS delivery

Patient PGx information was made available to study providers through an EHR‐integrated PGx CDS system developed by UCM that has been extensively described previously.^31^ The system delivered PGx information as a dynamic report containing a description of the patient’s result, prescribing recommendations, and references to original research. On one page of the system, providers could view a comprehensive list of all PGx‐annotated medication results. Additionally, each medication result was annotated with a CDS summary describing the PGx information and prescribing guidance. Each PGx CDS was categorized using a “traffic light” iconography: red indicated a genomic contraindication, yellow suggested caution due to a heightened risk of toxicity or inefficacy, and green denoted genomic compatibility.

The PGx CDS system was integrated into the EHRs of all three institutions as a single sign‐on system, accessible to enrolled providers via an internal browser within the EHR workspace. Enrolled inpatient care team members were notified of the availability of PGx results via a native EHR alert, which was triggered upon: (i) opening a patient’s chart; (ii) entering an order for a PGx‐annotated medication (specific to each patient); (iii) pharmacist verification of an order for a PGx‐annotated medication. These alerts did not discriminate based on the PGx results (i.e., they fired similarly for green, yellow, and red lights). After receiving an alert, providers could decide whether to access the PGx CDS by clicking on an embedded internal hyperlink. Alternatively, enrolled providers could also access the PGx CDS dashboard via a button on their EHR interface. Non‐enrolled providers did not receive any EHR alerts and did not have the PGx CDS Dashboard button enabled. Treatment decisions were at the sole discretion of treating providers. Because of feedback that providers were being “over‐alerted,” the open chart alert was discontinued on January 15, 2020.

### Data collection

Patient demographics were collected via survey at enrollment. Baseline number of medical comorbidities was collected using the International Classification of Diseases codes on the patient’s problem list. We collected medications via manual chart review. Medication lists were verified by independent review (by a separate research coordinator than the original recorder) for 10% of all charts. The discrepancy rate was so exceedingly low that the study team deemed additional chart verifications unwarranted.

### Outcome measures

For each admission, we identified the enrolled providers participating in the patient’s care. We captured prior‐to‐admission (PTA) medications (those each patient was taking at home at the time of admission), medications prescribed over the course of the admission (including time of ordering), and discharge medications. For PGx‐annotated medications, we recorded each medication’s PGx signal (light color). We captured whether enrolled providers accessed the PGx CDS system during each evaluable admission, defined as whether a provider “opened” a PGx CDS or entered the comprehensive PGx list result page during the admission (recording each time of accession).

The primary unit of analysis was evaluable admissions, defined as any inpatient admission of an enrolled, genotyped patient (whose results were available within the PGx CDS system) who was cared for by at least one study‐enrolled provider for at least 24 hours. The primary end point, PGx “discordant” medication prescribing, was quantified as the percentage of genomically cautionary (PGx yellow light) and genomically contraindicated (PGx red light) medications prescribed over an evaluable hospitalization. The independent variable was a binary measure of whether any enrolled member of the patient’s care team opened the PGx CDS system during the admission. All admission and readmission data were collected through May 31, 2021. Our primary hypothesis was that care teams that used the PGx CDS system would prescribe yellow/red PGx medications at lower rates compared to care teams that did not use the PGx CDS system.

There were two secondary hypotheses. The first was that care teams that used the PGx CDS system would discharge patients with a lower percentage of yellow/red medications (out of total number of discharge medications) compared to care teams that did not use the PGx CDS system. The second secondary hypothesis was that the percentage of PGx yellow/red discharge medications would predict a patient’s likelihood of being readmitted within 90 days of discharge. The independent variable was the percentage of PGx yellow/red discharge medications out of a patient’s total number of discharge medications.

### Statistical analyses

We performed descriptive statistics of patients and providers. This included frequencies and percentages for categorical variables and medians, ranges, and intervals for continuous variables. We performed descriptive statistics of PGx CDS use, including the time of CDS utilization, and descriptive statistics of prescribing, including percentages of red/yellow/green medications. We excluded from the per‐protocol analysis patients without any readmissions. We excluded admissions for which care teams did not include an enrolled provider and admissions for which the patient was not yet genotyped, as those care teams did not have an opportunity to access PGx results. We also excluded admissions < 24 hours in length, as those were typically observational admissions with minimal prescribing. For the secondary analysis on the likelihood of readmission, we excluded admissions that took place within the final 90 days of the data collection period for which no readmission had yet occurred, since those admissions were not able to be followed for a full 90 days; this excluded 55 admissions for this subanalysis.

For each hypothesis, we conducted univariate linear regressions of the outcome variable as a function of the pre‐defined independent variable. Statistics were performed using R (version 4.4.1, R Foundation for Statistical Computing, Vienna, Austria).

## RESULTS

### Patient and provider characteristics

In total, 531 patients were recruited, 67.0% of whom were recruited from UCM, 18.8% from NW, and 14.1% from UIC (**Table** **1**). 187 (35.2%) of patients were subsequently readmitted to inpatient medicine services (75.4% UCM, 17.6% NW, and 7.0% UIC), with a median of 2 (range 1–17) evaluable readmissions per readmitted patient during the course of the study. All readmitted patients self‐identified as Black or African American. In total, 113 (60.4%) were women. Readmitted patients had a median baseline of 14 prescriptions [range: 1–35] and 22 diagnoses [range: 1–84].

**Table 1:** Patient and provider characteristics across the multi‐institutional study

Patient characteristics^a^	Enrolled patients	UCM patients	NW patients	UIC patients	Readmitted patients
Total, *N* (%)	531 (100%)	356 (67.0%)	100 (18.8%)	75 (14.1%)	187 (35.2%)
Median age at enrollment [range]	57 [19–93]	58 [19–93]	56 [19–83]	52 [21–87]	55 [19–88]
Sex, *N* (%)					
Male	202 (38.0%)	145 (40.7%)	32 (32.0%)	50 (66.7%)	74 (39.6%)
Female	329 (62.0%)	211 (59.3%)	68 (68.0%)	25 (33.3%)	113 (60.4%)
Median medications at enrollment [range]	11 [1–35]	11 [1–35]	13 [1–33]	8 [1–24]	14 [1–35]
1–5	58 (10.9%)	31 (8.7%)	7 (7.0%)	20 (26.7%)	9 (4.8%)
6–10	184 (34.7%)	127 (35.7%)	26 (26.0%)	31 (41.3%)	46 (24.6%)
11–20	239 (45.0%)	165 (46.3%)	52 (52.0%)	22 (29.3%)	103 (55.1%)
21+	50 (9.4%)	33 (9.3%)	15 (15.0%)	2 (2.7%)	29 (15.5%)
Median diagnoses at enrollment [range]	20 [1–84]	23.5 [4–84]	14 [1–69]	12 [33–35]	22 [1–84]
1–10	91 (17.1%)	29 (8.1%)	35 (35.0%)	27 (36.0%)	20 (10.7%)
11–20	186 (35.0%)	113 (31.7%)	38 (38.0%)	35 (46.7%)	65 (34.8%)
21–30	139 (26.2%)	111 (31.2%)	19 (19.0%)	9 (12.0%)	54 (28.9%)
31–40	60 (11.3%)	51 (14.3%)	5 (5.0%)	4 (5.3%)	18 (9.6%)
41+	55 (10.4%)	52 (14.6%)	3 (3.0%)	0 (0.0%)	30 (16.0%)
Number of readmissions^b^					Median = 2 [range 1–17]
0	344 (64.8%)	215 (60.4%)	67 (67.0%)	62 (82.7%)	0 (0%)
1	89 (16.8%)	64 (18.0%)	15 (15.0%)	10 (13.3%)	89 (47.6%)
2–3	57 (10.7%)	44 (12.4%)	12 (12.0%)	1 (1.3%)	57 (30.5%)
4–5	22 (4.1%)	18 (5.1%)	2 (2.0%)	2 (2.7%)	22 (11.8%)
6+	19 (3.6%)	15 (4.2%)	4 (4.0%)	0 (0.0%)	19 (10.1%)

Provider characteristics	Overall providers	UCM providers	NW providers	UIC providers
Total, *N* (%)^c^	188 (100%)	99 (52.7%)	65 (34.6%)	24 (12.8%)
Provider type, *N* (%)^d^				
Physician	120 (63.8%)	62 (62.6%)	46 (70.8%)	12 (50.0%)
Pharmacist	42 (22.3%)	18 (18.2%)	12 (18.5%)	12 (50.0%)
Physician assistant	12 (6.4%)	6 (6.1%)	6 (9.2%)	0 (0.0%)
Advanced practice nurse	14 (7.4%)	13 (13.1%)	1 (1.5%)	0 (0.0%)
Median years of practice [range]	3 [0–36]	2 [0–20]	3 [0–20]	11.5 [1–36]
0–3	101 (53.7%)	63 (63.6%)	35 (53.8%)	3 (12.5%)
4–6	37 (19.7%)	19 (19.2%)	16 (24.6%)	2 (8.3%)
7–9	17 (9.0%)	9 (9.1%)	5 (7.7%)	3 (12.5%)
10+	33 (17.6%)	8 (8.1%)	9 (13.8%)	16 (66.7%)
Median admissions per enrolled provider [standard deviation]	5 [1–148]	12 [1–148]	3 [1–14]	1 [1–10]
1	36 (19.1%)	7 (7.1%)	16 (24.6%)	13 (54.2%)
2–5	64 (34.0%)	16 (16.2%)	38 (58.5%)	10 (41.7%)
6–10	33 (17.6%)	22 (22.2%)	10 (15.4%)	1 (4.2%)
11+	55 (29.3%)	54 (54.5%)	1 (1.5%)	0 (0.0%)

^a^ Per National Institute on Minority Health and Health Disparities funding parameters, all patients had to self‐identify as Black or African American to participate in this trial. Thus, race and ethnicity are not reported in tabular format. Of note, during enrollment, 2 patients reported their race as American Indian/Alaskan Native, 1 as White, 10 as More Than One Race/Other, and 2 as Unknown (all at UCM). Similarly, 5 patients reported their ethnicity as Hispanic or Latino, and 1 as Unknown.

^b^ Includes only evaluable admissions.

^c^ An additional 84 enrolled providers did not care for an enrolled patient over the trial period; thus, they did not have the opportunity to access a PGx result and were excluded from **Table** **1**.

^d^ “Physicians” indicate attending physicians only. Physicians in training (i.e., residents and fellows), although they may have been involved in the care of study patients, were not eligible for enrollment in this trial; they did not have access to PGx results.

In total, 188 (69.1%) of 272 enrolled providers cared for an enrolled patient over the study period (**Table** **1**); 120 (63.8%) were physicians, 42 (22.3%) were pharmacists, 12 (6.4%) were physician assistants, and 14 (7.4%) were advanced practice nurses. In total, 99 (52.7%) of providers were employed by UCM, 65 (34.6%) by NW, and 24 (12.8%) by UIC. Additional provider demographics were previously reported.^33^

### Admission characteristics

Enrolled patients experienced 518 evaluable admissions; 417 (80.5%) at UCM, 80 (15.4%) at NW, and 21 (4.1%) at UIC. Admission details are shown in **Table** **2**.

**Table 2:** Prescribing patterns and prevalence of pharmacogenomically annotated prescriptions associated with inpatient hospitalizations

Admission characteristics	Value
Total, *N* a	518
University of Chicago Medical Center, *N* (%)	417 (80.5%)
Northwestern Memorial Hospital, *N* (%)	80 (15.4%)
University of Illinois Hospital & Health Sciences System, *N* (%)	21 (4.1%)
Pharmacogenomic clinical decision support use during admission, *N* (%)	260 (50.2%)
Prior‐to‐admission (PTA) medications	
Median PTA medications per admission [range]	14 [range 0–46]
Median genomically annotated PTA medications per admission [range]	3 [range 0–12]
Admissions with genomically annotated PTA medications, *N* (%)	486 (93.8%)
Mean percentage of “Green” PTA medications [standard deviation (SD)]	19.4% [SD = 12.2%]
Mean percentage of “Yellow” PTA medications [SD]	4.6% [SD = 5.7%]^b^
Mean Percentage of “Red” PTA Medications [SD]	0.2% [SD = 1.1%]^b^
Hospitalization medications	
Median hospitalization medications per admission [range]	6 [range 0–94]
Median genomically annotated medications per admission [range]	0 [range 0–9]^c^
Admissions with genomically annotated hospitalization medications, *N* (%)	248 (47.9%)
Mean percentage of “Green” hospitalization medications [SD]	7.6% [SD = 13.3%]
Mean percentage of “Yellow” hospitalization medications [SD]	3.6% [SD = 10.6%]^d^
Mean percentage of “Red” hospitalization medications [SD]	0.0% [SD = 0.0%]^d^
Discharge medications	
Median discharge medications per admission [range]	13 [range 0–36]
Median genomically annotated medications per admission [range]	3 [range 0–12]
Admissions with genomically annotated discharge medications, *N* (%)	473 (91.3%)
Mean percentage of “Green” discharge medications [SD]	18.4% [SD = 11.9%]
Mean percentage of “Yellow” discharge medications [SD]	4.6% [SD = 5.6%]^e^
Mean percentage of “Red” discharge medications [SD]	0.1% [SD = 0.8%]^e^
Median percentage of PTA medications continued at discharge [range]	90.0% [range 0–100%]
Median Percentage of Genomically annotated PTA medications continued at discharge [range]	100.0% [range 0–100%]

^a^ 72 admissions were not evaluable because the care team did not include an enrolled provider. Additionally, 42 admissions were not evaluable due to data collection interruptions during the COVID‐19 pandemic.

^b^ Out of 486 admissions with genomically annotated PTA medications, 251 admissions (51.6%) had 367 PTA medications categorized as yellow and 16 admissions (3.3%) had 16 PTA medications categorized as red (one each).

^c^ Out of 518 admissions overall, 248 admissions (47.9%) had 377 genomically annotated prescriptions.

^d^ Out of 248 admissions with genomically annotated hospitalization prescribing, 98 admissions (39.5%) had 108 prescribed medications categorized as yellow. 0 hospitalization medications were categorized as red.

^e^ Out of 473 admissions with genomically annotated discharge prescribing, 251 admissions (53.1%) had 370 discharge medications categorized as yellow, and 10 admissions (2.1%) had 10 discharge medications categorized as red (one each).

### Prescribing characteristics

**Figure 1:**
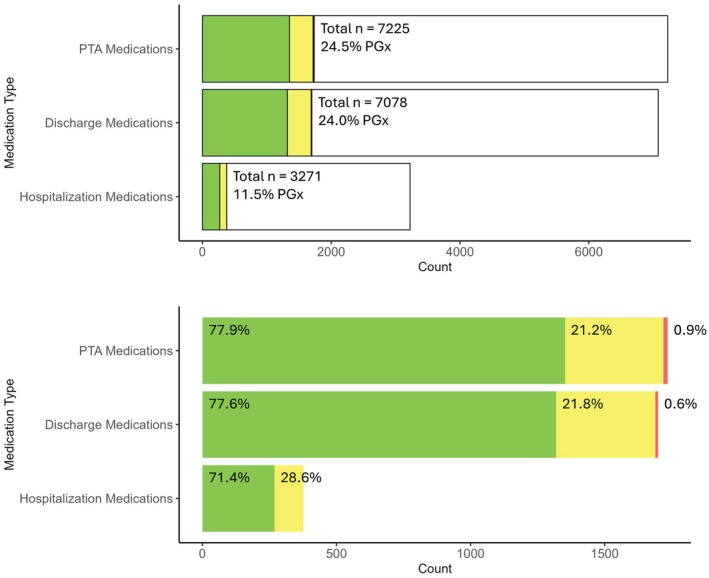
Pharmacogenomic results of prior‐to‐admission (PTA) medications, discharge medications, and hospitalization medications of african american inpatients. Top panel shows genomically annotated (PGx = “Pharmacogenomic”) medications as a percentage of all medications. Bottom panel shows only PGx medications and their genomic risk categorizations (green as genomically compatible, yellow as increased caution, and red as genomically contraindicated).

Patients were admitted with a median of 14 PTA medications [range: 0–46] (**Table** **2**), and only 32 (6.2%) admissions included patients without any PGx‐annotated PTA medications. An average 19.4% [standard deviation (SD) = 12.2%] of PTA medications were categorized as green lights (i.e., genomically favorable), an average 4.6% [SD = 5.7%] of PTA medications were categorized as yellow lights (i.e., genomically cautionary due to risk of reduced efficacy or increased toxicity), and an average 0.2% [SD = 1.1%] of PTA medications were categorized as red lights (i.e., genomically contraindicated). Overall, of the total 7,225 PTA medications, 1,735 (24.5%) were PGx‐annotated (**Figure** **1**). Of these, 1,352 (77.9%) were categorized as green lights, 367 (21.2%) as yellow lights, and 16 (0.9%) as red lights.

During admissions, patients were prescribed a median of 6 medications [range: 0–94] (**Table** **2**). In 270 (52.1%) admissions, patients were not prescribed any PGx‐annotated medications. Across all evaluable admissions, an average of 7.6% [SD = 13.3%] of all prescribed medications were categorized as green lights, an average of 3.6% [SD = 10.6%] of all prescribed medications were categorized as yellow lights, and no prescribed medications during admissions, for the duration of the entire study, were categorized as red lights. Overall, patients were prescribed 3,271 medications during their hospitalization; 377 (11.5%) were PGx‐annotated (**Figure** **1**). Of these, 269 (71.4%) were categorized as green lights and 108 (28.6%) as yellow lights. The most frequently prescribed hospitalization medications were pantoprazole (101 prescriptions, categorized as green for 30.2% of patients; **Table** **3**), tramadol (36 prescriptions, green for 77.4%), and oxycodone (30 prescriptions, green for 77.4%). **Table** **S1** lists all genomically cautionary (yellow) hospitalization medications prescribed during evaluable admissions over the trial, regardless of whether care teams used the PGx system. Of note, a modest number (13 patients) of the 57 total study patients who received genomically cautionary hospitalization medications accounted for 50.9% (55/108) of these discordant prescriptions. This reflects a pattern where the same medications would often be repeatedly used/continued for the same frequently readmitted patients across several hospitalizations.

**Table 3:** Top 10 most frequently prescribed genomically annotated medications across all evaluable admissions and genomic risk categorization among patients with results

Drug	Prescribing frequency across all evaluable admissions (*n* = 518)	Genomic risk categorization among all genotyped patients (*n* = 483)^a^		
		Green (%)	Yellow (%)	Red (%)
Pantoprazole	101	30.2	57.3	0
Tramadol	36	77.4	8.3	1.0
Oxycodone	30	77.4	9.3	0
Hydralazine	28	75.6	20.7	0
Budesonide	24	91.5	7.7	0
Morphine	23	93.6	5.6	0
Aspirin	17	81.0	16.4	0
Fluticasone propionate	17	91.5	7.7	0
Ibuprofen	14	94.6	3.5%	0
Carvedilol	13	24.8	45.3%	0

^a^ For all medications in Table 3, green, yellow, and red percentages do not add to 100% because there was a small percentage of patients for whom no genomic annotation was available for that specific medication, usually due to indeterminate genotype calls.

Patients were discharged with a median of 13 medications [range: 0–36] (**Table** **2**). In only 45 (8.7%) admissions, patients were not discharged with any PGx‐annotated medications. Across all hospital discharges, an average of 18.4% [SD = 11.9%] of discharge medications were categorized as green lights, an average of 4.6% [SD = 5.6%] of discharge medications were categorized as yellow lights, and an average of 0.1% [SD = 0.8%] of discharge medications were categorized as red lights. Overall, of the total 7,078 discharge medications, 1,699 (24.0%) were PGx‐annotated medications (**Figure** **1**). Of these, 1,319 (77.6%) were categorized as green lights, 370 (21.8%) as yellow lights, and 10 (0.6%) as red lights.

Notably, a median 90.0% [range: 0–100%] of PTA medications were continued at discharge (**Table** **2**). In 217 of 253 admissions (85.8%) where the patient was admitted with at least one yellow PTA medication, and in 10 out of 16 admissions (62.5%) where the patient was admitted with at least one red PTA medication, the care team discharged the patient on the same yellow and/or red medications.

### Differences in hospitalization and discharge prescribing among teams that did or did not use the PGx CDS system

The primary hypothesis of the ACCOuNT Trial was that care teams that used the PGx CDS system would prescribe yellow/red medications at lower rates compared to care teams that did not use PGx CDS. Among admissions managed by teams that used the PGx CDS, 3.9% [SD = 11.4%] of hospitalization medications were yellow lights, compared to 3.3% [SD = 9.7%] among teams that did not use the PGx CDS system (**Table** **4**; *P* > 0.05).

**Table 4:** Prescribing patterns between care teams that used pharmacogenomic clinical decision support (PGx CDS) and care teams that did not use PGx CDS

	Care teams that used PGx CDS	Care teams that did not use PGx CDS	*P*‐value
Prescribing of yellow/red hospitalization medications	3.9% yellow/red hospitalization medications	3.3% yellow/red hospitalization medications	*P* = 0.53
Prescribing of yellow/red discharge medications^a^	4.0% yellow/red discharge medications	5.4% yellow/red discharge medications	*P* < 0.01

^a^ The percentage of yellow/red prior‐to‐admission (PTA) medications differed significantly between admissions within care teams that used the pharmacogenomic clinical decision support (PGx CDS) system and admissions within care teams that did not use the PGx CDS system (4.2% vs. 5.3%, respectively; *P* < 0.05). When accounting for this significant difference in yellow/red PTA medications, the percentage change in yellow/red medications from PTA to discharge did not differ significantly between teams that used the PGx CDS system and teams that did not use it (−0.2% vs. 0.1%, *P* > 0.05).

A secondary hypothesis of the ACCOuNT Trial was that care teams that used the PGx CDS system would discharge patients on yellow/red medications at lower rates compared to care teams that did not use PGx CDS. Among admissions managed by teams that used PGx CDS, 4.0% [SD = 5.1%] of discharge medications were categorized as yellow/red lights, compared to 5.4% [SD = 6.3%] among teams that did not use PGx CDS (**Table** **4**; *P* < 0.01). However, the percentage of yellow/red PTA medications also differed significantly between admissions with care teams that used the PGx CDS system and admissions with care teams that did not use the PGx CDS system (4.2% vs. 5.3%, *P* < 0.05). After accounting for this significant difference in yellow/red PTA medications, the percentage change in yellow/red medications from PTA to discharge did not differ significantly between teams that used the PGx CDS system and teams that did not use it (−0.2% vs. 0.1%, *P* > 0.05).

### Predictiveness of cautionary or contraindicated discharge medications on likelihood of 90‐day readmission

Overall, patients were readmitted within 90 days in 196 (42.4%) admissions. We found no statistically significant relationship between the percentage of yellow/red discharge medications and the likelihood of readmission within 90 days (*P* > 0.05).

### PGx CDS utilization and timing

PGx results were available for all 187 (100.0%) readmitted patients. Care teams accessed the PGx CDS system during 260 (50.2%) out of 518 total evaluable admissions. Among the 248 (47.9%) admissions with PGx‐annotated hospitalization medications, care teams accessed the PGx CDS system on 144 (58.1%) admissions. Across the course of the study, care teams accessed the PGx CDS system during 83.7% (36/43) of evaluable admissions in 2017, 76.9% (80/104) of admissions in 2018, 49.5% (50/101) of admissions in 2019, 33.5% (55/164) of admissions in 2020, and 36.8% (39/106) of admissions in 2021. Some predictors of PGx CDS utilization across the trial, including differences in utilization by study site, have been previously described.^33^

**Figure 2:**
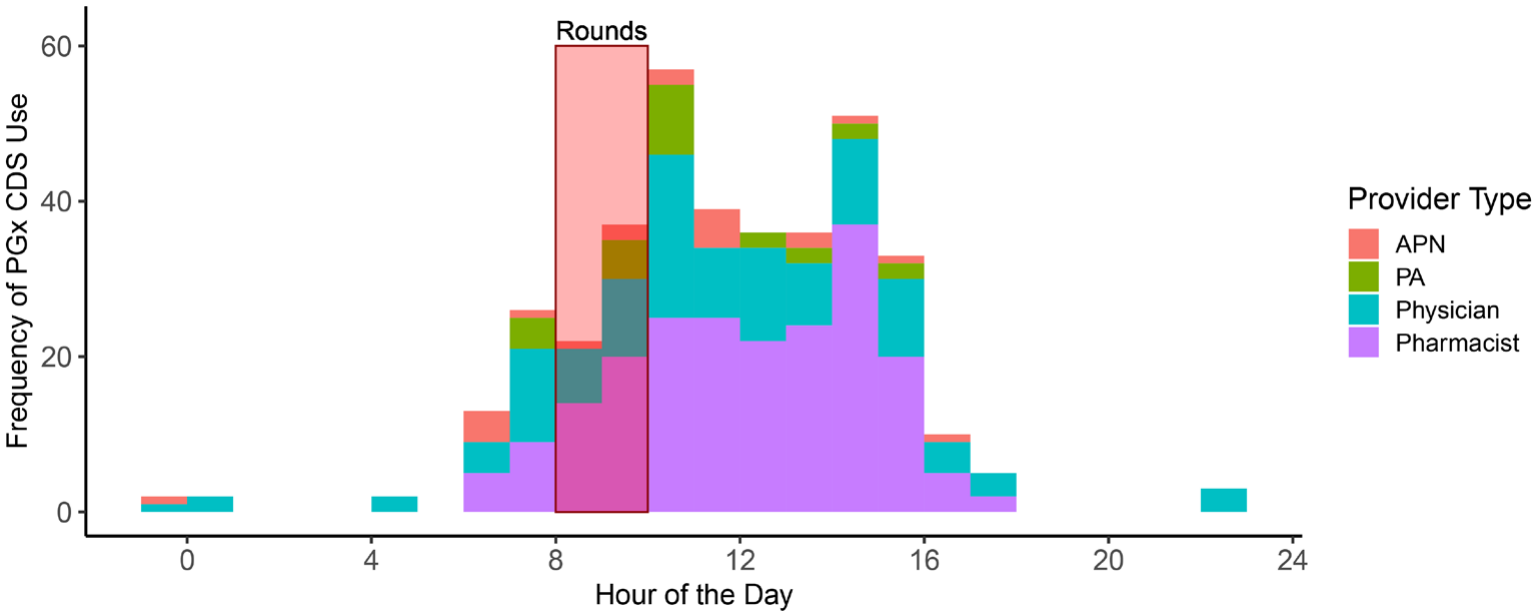
Timing of pharmacogenomic clinical decision support (PGx CDS) use across the day, by provider role. The red box labeled “rounds” indicate when patient rounding typically occurs on inpatient medicine services. APN, advanced practice nurse; PA, physician assistant.

**Figure 3:**
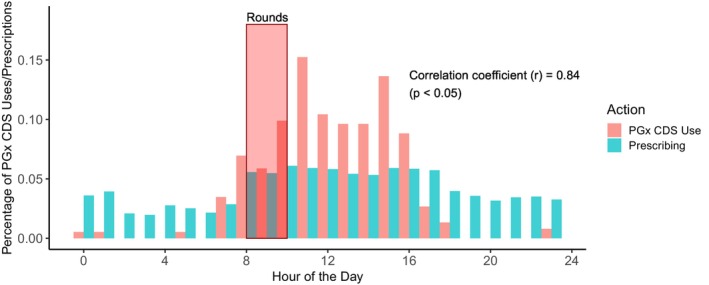
Pharmacogenomic clinical decision support (PGx CDS) use relative to prescribing across the day. The red box labeled “rounds” indicate when patient rounding typically occurs on inpatient medicine services.

In total, 347 of 374 (92.8%) PGx CDS system accessions occurred between the hours of 8 a.m. and 5 p.m. (**Figure** **2**). 307 (82.1%) PGx CDS system accessions occurred after 10 am, when rounding typically ends on inpatient medicine services. Notably, total PGx CDS accession timing across the day significantly correlated with prescribing timing, which was defined as when a provider ordered a medication in the EHR (**Figure** **3**; *r* = 0.84, *P* < 0.05).

## DISCUSSION

To our knowledge, this is the first study to report results from a multi‐institutional, prospective PGx implementation trial focused exclusively on underrepresented minority patients. Surprisingly, no genomically contraindicated medications and a low percentage of genomically cautionary medications were prescribed in relation to the inpatient hospitalizations within our study. Perhaps because of this, and despite a 50.2% PGx CDS utilization rate, we found that utilization did not significantly reduce PGx cautionary or contraindicated (“discordant”) prescribing among African American patients in the inpatient setting. Nevertheless, we found that inpatient care teams repeatedly utilized PGx CDS, most commonly as a form of prescribing reassurance for genomically concordant medications. Finally, we demonstrated the utility of an EMR‐embedded PGx results system that deployed consistent and scalable PGx CDS across multiple hospitals, representing a key informatics solution for PGx implementation. These findings invite further investigation into the utility of PGx information among underrepresented and frequently hospitalized patients.

Several factors may contextualize these results. First, self‐identified African American patients are underrepresented within genomic studies that inform current PGx CDS recommendations.^34^, ^35^ This factor partially motivated the ACCOuNT study, and given the low number of genomically contraindicated medications prescribed throughout this trial, likely reflects our field’s potentially incomplete knowledge of actionable high‐risk PGx variants in populations of non‐European ancestry. Said another way, if additional actionable variants were ‘known’ and included in our testing platforms and CDS recommendations that were exclusively or more likely to be present in individuals of African ancestry, then the prevalence of “PGx discordant” medications in this study (and the overall utility) may have been higher. This emphasizes the imperative for ongoing PGx discovery work in underrepresented populations, including the likely need for future sequencing efforts to identify currently undiscovered variants.

The question of PGx’s value for inpatient medicine settings is further illuminated by considering the medications that are prescribed during inpatient stays, and how those align with current PGx guidelines. Most major guideline‐based PGx medications are “outpatient” drugs (like statins, chemotherapies, antidepressants), given chronically to patients. In contrast, inpatient medications are more commonly “short term” medications like intravenous antibiotics, anticoagulants, and antihypertensives, which currently have generally less PGx guidance. Inpatient physicians seemed to be quite reluctant to change perhaps longstanding “prior‐to‐admission” medications. This is understandable, as these physicians are not the providers that initiated these medications, and furthermore, if patients are stable on these medications, then there would be little impetus to change that which is working. The pattern we observed where genomically cautionary medications would often be repeatedly used/continued for a patient across multiple hospitalizations likely reflects the idea that if providers cautiously prescribed a “yellow light” medication and observed that the medication was successfully treating the patient’s condition, then there would be little rationale to change/revisit their prescribing decision during that admission or subsequent readmissions. Our results thus suggest that PGx utility is perhaps better measured over a lifespan, rather than within the confines of a single (or even several) inpatient medicine admissions. Inpatient‐specific PGx CDS may yield greater utility if deployed in targeted, high‐value clinical contexts such as gastric ulcer management, palliative care/pain management consultations, and post‐cardiovascular catheterization procedures, as these are frequent causes for admission that are treated with commonly utilized medications that have clear and actionable PGx evidence. These findings, along with the previously mentioned fact that most PGx annotations for the medications used in this study were concordant (“green lights”), almost certainly explain why this study was unable to find that PGx CDS use decreased genomically discordant prescribing.

The ACCOuNT trial’s ~50% PGx CDS utilization rate interestingly mirrors the ~50% of admissions where genomically annotated hospitalization prescribing occurred. While we did not discover a significant relationship between genomically annotated prescribing and PGx CDS utilization, we surmise that this result reflects a possible provider “learning effect.” Inpatient care teams found annotated PGx information about half of the time when they accessed the PGx CDS system. Even when they found PGx annotations, the prescribing recommendations for their desired drugs consisted mostly of genomically compatible (“green light”) signals, with few cautionary signals and very rare PGx contraindications. Our personal learnings from care teams in this and other trials affirm this idea of provider learning, which may be especially relevant for this study’s frequently admitted patient population.^36^ About half of all readmitted patients were admitted multiple times to the same inpatient medicine service, often staffed by the same providers. In these cases, providers may have recalled the PGx information (or lack thereof) from the prior admission and chose to not reference the PGx CDS again.

ACCOuNT was notable for its approach to solving the complex challenges of scalable multi‐institutional PGx CDS implementation. As mentioned, all three institutions in this trial implemented the same EHR‐integrated PGx CDS system, a major accomplishment that enabled us to study standardized PGx CDS utilization across multiple institutions. As similarly extensible technologies proliferate, we anticipate growing opportunities to study scalable PGx CDS systems, which may improve our collective ability to detect differences in PGx clinical outcomes.

Even before the completion of the ACCOuNT trial, we received informal feedback from providers at hospital faculty meetings about the need for thoughtful and parsimonious use of interruptive alerts, which led to our team discontinuing the “open chart” alert mid‐trial. In the time since, our institution has pivoted toward the selective use of interruptive alerts only for cautionary (yellow) and incompatible (red) medications, thus intervening in the clinical workflow only when there is highly important information. However, the downside to such an approach is that it deprives providers of the possible “prescribing reassurance” that a “green light” PGx CDS can provide. We found intriguing evidence for this latter idea. Across all three institutions, > 80% of PGx CDS accessions occurred after 10 am, presumably after inpatient rounds where most patient care plans were determined. We also found that the time of PGx CDS use significantly correlated with the time of medication ordering. Collectively, these results suggest that care teams repeatedly utilized PGx information as prescribing reassurance, where providers (usually pharmacists) consulted and referenced the PGx CDS system as they executed a patient’s care plan after it was determined on rounds. While such reassurance did not lead to detectable changes in the care plan—as most PGx results were genomically compatible—this is nonetheless a significant insight into how PGx CDS may be most useful. The value of PGx as prescribing reassurance has been discussed in some qualitative and survey research,^37^, ^38^ but has never before been measured in a prospective PGx implementation trial. Additionally, the value of prescribing reassurance may not be easily captured using existing PGx implementation measures such as genomically contraindicated prescribing. Prescribing reassurance by definition does not change the original prescribing decision. As such, the impact of prescribing reassurance on PGx utilization may be insufficiently explored in PGx trials that are focused on—and powered for—reducing “high risk” prescribing.

This study had limitations. Less than half of all patients enrolled were readmitted, limiting the number of evaluable admissions and decreasing theoretical power to demonstrate a difference in discordant prescribing. This may reflect the challenge of PGx deployment in underrepresented and often underserved patient populations, who may experience unique access‐to‐care constraints that could limit opportunities for PGx‐guided medication management. Furthermore, concerns for out‐of‐pocket medication costs may override concerns for genomic incompatibility, especially if the majority of PGx guidance is only “cautionary” rather than “incompatible” (as was the case here). Future studies may consider high‐value PGx deployments in targeted clinical contexts (gastrointestinal ulcers/bleeding, palliative care/pain management, post‐cardiac catheterization, and likely other settings) as a more judicious use of limited healthcare resources. Additionally, patients who are less likely to re‐engage within a given health system where PGx testing might be initially ordered, and those who may be unlikely to utilize regular, outpatient, and preventative medical care settings, could be expected to realize relatively fewer benefits from PGx testing, given our learnings from this study. Second, we utilized self‐identified race to determine patient eligibility. Race is a social construct without intrinsic scientific or biological meaning.^39^, ^40^ While ACCOuNT sought to understand the utility of PGx for patients whose genotypes are underrepresented in genomic studies, we acknowledge that race is an imperfect proxy for capturing these variants. Other prior smaller studies have also examined similar themes in underrepresented populations,^2^, ^3^, ^4^ and another ongoing trial is also weighing these important questions about the applicability of genomic findings to specific groups.^26^ However, to date, no prior published study has included the scope and size of our current effort. Separately, prescribers such as residents, emergency medicine physicians, and other consultants were not given access to PGx CDS results within this study; if these providers had been included, it is possible that larger effects on prescribing would have been detectable. Other trials are in fact currently looking specifically at the Emergency Department as a possible PGx utility setting.^41^ Patients were also not given direct access to their own PGx results in this study, an intriguing area of intervention and patient self‐advocacy that we have since begun to explore.^42^ Penultimately, we were not powered to study medication‐related adverse events. Instead, our findings focus on the key intermediate step of genomically cautionary or contraindicated prescribing, which may inform later studies focusing on adverse drug reactions in underrepresented and inpatient populations. Finally, ACCOuNT enrolled and evaluated patients during the COVID‐19 pandemic, which likely limited the total number and type of admissions. Despite this challenge, ACCOuNT successfully uncovered novel insights toward the use and relevance of PGx CDS among underrepresented inpatients.

In conclusion, in the first results of a multi‐institutional, prospective PGx implementation trial focusing on the care of underrepresented inpatients, we successfully scaled a PGx CDS solution that informed the care of underrepresented, frequently hospitalized patients. We found that existing PGx information had modest relevance to the care of African American inpatients; we were unable to demonstrate that PGx CDS deployment decreased genomically discordant prescribing. Nevertheless, we observed a high correlation between PGx accession and time of prescribing, and we found consistent support for the use of concordant PGx CDS as prescribing reassurance.

## FUNDING

This study was funded by U54MD010723, The National Institutes of Health (NIH).

## CONFLICT OF INTEREST

P.H.O. reports receiving honoraria for service as part of the NIH IGNITE network data safety monitoring board, which had no connection to this work. P.H.O. also reports having received prior payments for legal consultative services involving pharmacogenomics. M.J.R. receives royalties related to *UGT1A1* genotyping that is unrelated to any genotyping that was performed in this work.

## DISCLAIMER

As an Associate Editor of *Clinical Pharmacology & Therapeutics*, Peter H. O’Donnell was not involved in the review or decision making processes for this article.

## AUTHOR CONTRIBUTIONS

Z.H. and P.H.O. wrote the manuscript. P.H.O., K.J.O., E.A.N., M.A.P., M.J.R., and D.O.M. designed the research. T.C., G.W.R., J.C.L., D.G., L.K.H., R.K., L.V.R., S.H., K.‐T.J.Y., and A.C. performed the research. Z.H., M.J., and P.H.O. analyzed the data.

## Supporting information


Table S1

## References

[1] Smith, D.M. , Wake, D.T. & Dunnenberger, H.M. Pharmacogenomic clinical decision support: a scoping review. Clin. Pharmacol. Ther. 113, 803–815 (2022). 35838358 10.1002/cpt.2711

[2] Ettienne, E.B. *et al*. Pharmacogenomics and opioid use disorder: clinical decision support in an African American cohort. J. Natl. Med. Assoc. 111, 674–681 (2019). 31676110 10.1016/j.jnma.2019.09.006 PMC8815013

[3] Lteif, C. , Eddy, E. , Terrell, J. , Cavallari, L.H. , Malaty, J. & Duarte, J.D. Feasibility of preemptive pharmacogenetic testing and improvement of medication treatment satisfaction among medically underserved patients. Clin. Transl. Sci. 17, e13692 (2024). 38013396 10.1111/cts.13692 PMC10772669

[4] J Sargent, L. *et al*. The translational approaches to personalized health collaborative: pharmacogenomics for African American older adults. Clin. Transl. Sci. 14, 437–444 (2021). 33026148 10.1111/cts.12885 PMC7993264

[5] Manzi, S.F. *et al*. Creating a scalable clinical pharmacogenomics service with automated interpretation and medical record result integration – experience from a pediatric tertiary care facility. J. Am. Med. Inform. Assoc. 24, 74–80 (2017). 27301749 10.1093/jamia/ocw052 PMC7654081

[6] Peterson, J.F. *et al*. Physician response to implementation of genotype‐tailored antiplatelet therapy. Clin. Pharmacol. Ther. 100, 67–74 (2016). 26693963 10.1002/cpt.331 PMC4899238

[7] Ubanyionwu, S. , Formea, C.M. , Anderson, B. , Wix, K. , Dierkhising, R. & Caraballo, P.J. Evaluation of prescriber responses to pharmacogenomics clinical decision support for thiopurine S‐methyltransferase testing. Am. J. Health‐Syst. Pharm. 75, 191–198 (2018). 29436466 10.2146/ajhp170280

[8] Gammal, R.S. *et al*. Pharmacogenetics for safe codeine use in sickle cell disease. Pediatrics 138, e20153479 (2016). 27335380 10.1542/peds.2015-3479 PMC4925073

[9] O’Donnell, P.H. *et al*. Pharmacogenomics‐based point‐of‐care clinical decision support significantly alters drug prescribing. Clin. Pharmacol. Ther. 102, 859–869 (2017). 28398598 10.1002/cpt.709 PMC5636653

[10] Bank PCD , Swen, J.J. , Schaap, R.D. , Klootwijk, D.B. , Baak, P.R. & Guchelaar, H.J. A pilot study of the implementation of pharmacogenomic pharmacist initiated pre‐emptive testing in primary care. Eur. J. Hum. Genet. 27, 1532–1541 (2019). 31227807 10.1038/s41431-019-0454-x PMC6777565

[11] Nutescu, E.A. *et al*. Feasibility of implementing a comprehensive warfarin pharmacogenetics service. Pharmacotherapy 33, 1156–1164 (2013). 23864527 10.1002/phar.1329 PMC3985126

[12] Wu, R.R. *et al*. Implementation, adoption, and utility of family health history risk assessment in diverse care settings: evaluating implementation processes and impact with an implementation framework. Genet. Med. 21, 331–338 (2019). 29875427 10.1038/s41436-018-0049-x PMC6281814

[13] Eadon, M.T. *et al*. The INGENIOUS trial: impact of pharmacogenetic testing on adverse events in a pragmatic clinical trial. Pharmacogenomics J. 23, 169–177 (2023). 37689822 10.1038/s41397-023-00315-w PMC10805517

[14] Greden, J.F. *et al*. Impact of pharmacogenomics on clinical outcomes in major depressive disorder in the GUIDED trial: a large, patient‐ and rater‐blinded, randomized, controlled study. J. Psychiatr. Res. 111, 59–67 (2019). 30677646 10.1016/j.jpsychires.2019.01.003

[15] Oslin, D.W. *et al*. Effect of pharmacogenomic testing for drug‐gene interactions on medication selection and remission of symptoms in major depressive disorder. JAMA 328, 151–161 (2022). 35819423 10.1001/jama.2022.9805 PMC9277497

[16] Cicali, E.J. *et al*. Challenges and lessons learned from clinical pharmacogenetic implementation of multiple gene–drug pairs across ambulatory care settings. Genet. Med. 21, 2264–2274 (2019). 30926959 10.1038/s41436-019-0500-7 PMC6768772

[17] Herr, T.M. , Peterson, J.F. , Rasmussen, L.V. , Caraballo, P.J. , Peissig, P.L. & Starren, J.B. Pharmacogenomic clinical decision support design and multi‐site process outcomes analysis in the eMERGE network. J. Am. Med. Inform. Assoc. 26, 143–148 (2019). 30590574 10.1093/jamia/ocy156 PMC6339514

[18] Dawes, M. *et al*. Introducing pharmacogenetic testing with clinical decision support into primary care: a feasibility study. CMAJ Open 4, E528–E534 (2016). 10.9778/cmajo.20150070 PMC5047800 27730116

[19] Zhang, H. , De, T. , Zhong, Y. & Perera, M.A. The advantages and challenges of diversity in pharmacogenomics: can minority populations bring us closer to implementation? Clin. Pharmacol. Ther. 106, 338–349 (2019). 31038731 10.1002/cpt.1491 PMC6663589

[20] Magavern, E.F. , Gurdasani, D. , Ng, F.L. & Lee, S.S.J. Health equality, race and pharmacogenomics. Br. J. Clin. Pharmacol. 88, 27–33 (2022). 34251046 10.1111/bcp.14983 PMC8752640

[21] Popejoy, A.B. & Fullerton, S.M. Genomics is failing on diversity. Nature 538, 161–164 (2016). 27734877 10.1038/538161a PMC5089703

[22] Friedman, P.N. *et al*. The ACCOuNT consortium: a model for the discovery, translation, and implementation of precision medicine in African Americans. Clin. Transl. Sci. 12, 209–217 (2019). 30592548 10.1111/cts.12608 PMC6510376

[23] Expired RFA‐MD‐15‐013: NIMHD transdisciplinary collaborative centers for health disparities research focused on precision medicine (U54). <https://grants.nih.gov/grants/guide/rfa‐files/RFA‐MD‐15‐013.html#_Section_III._Eligibility>. Accessed February 17, 2025. 10.18865/ed.30.S1.135 PMC7138448 32269454

[24] Ginsburg, G.S. *et al*. Establishing the value of genomics in medicine – the IGNITE pragmatic trials network. Genet. Med. 23, 1185–1191 (2021). 33782552 10.1038/s41436-021-01118-9 PMC8263480

[25] Rasmussen‐Torvik, L.J. *et al*. Design and anticipated outcomes of the eMERGE‐PGx project: a multicenter pilot for preemptive pharmacogenomics in electronic health record systems. Clin. Pharmacol. Ther. 96, 482–489 (2014). 24960519 10.1038/clpt.2014.137 PMC4169732

[26] Eadon, M.T. *et al*. Design and rationale of GUARDD‐US: a pragmatic, randomized trial of genetic testing for APOL1 and pharmacogenomic predictors of antihypertensive efficacy in patients with hypertension. Contemp. Clin. Trials 119, 106813 (2022). 35660539 10.1016/j.cct.2022.106813 PMC9928488

[27] Schneider, T.M. *et al*. Multi‐institutional implementation of clinical decision support for APOL1, NAT2, and YEATS4 genotyping in antihypertensive management. J. Pers. Med. 11, 480 (2021). 34071920 10.3390/jpm11060480 PMC8226809

[28] Blagec, K. *et al*. Implementing pharmacogenomics decision support across seven European countries: the ubiquitous pharmacogenomics (U‐PGx) project. J. Am. Med. Inform. Assoc. 25, 893–898 (2018). 29444243 10.1093/jamia/ocy005 PMC6016647

[29] Swen, J.J. *et al*. A 12‐gene pharmacogenetic panel to prevent adverse drug reactions: an open‐label, multicentre, controlled, cluster‐randomised crossover implementation study. Lancet 401, 347–356 (2023). 36739136 10.1016/S0140-6736(22)01841-4

[30] Caudle, K.E. *et al*. Standardizing terms for clinical pharmacogenetic test results: consensus terms from the clinical pharmacogenetics implementation consortium (CPIC). Genet. Med. 19, 215–223 (2017). 27441996 10.1038/gim.2016.87 PMC5253119

[31] Danahey, K. *et al*. Simplifying the use of pharmacogenomics in clinical practice: building the genomic prescribing system. J. Biomed. Inform. 75, 110–121 (2017). 28963061 10.1016/j.jbi.2017.09.012

[32] Tang, N.Y. *et al*. Validation of a large custom‐designed pharmacogenomics panel on an Array genotyping platform. J. Appl. Lab. Med. 6, 1505–1516 (2021). 34263311 10.1093/jalm/jfab056 PMC8561785

[33] Huang, Z. *et al*. Care team attributes predict likelihood of utilizing pharmacogenomic information during inpatient prescribing. Clin. Transl. Sci. 18, e70193 (2025). 40259529 10.1111/cts.70193 PMC12011642

[34] Pirmohamed, M. Pharmacogenomics: current status and future perspectives. Nat. Rev. Genet. 24, 350–362 (2023). 36707729 10.1038/s41576-022-00572-8

[35] Mills, M.C. & Rahal, C. The GWAS diversity monitor tracks diversity by disease in real time. Nat. Genet. 52, 242–243 (2020). 32139905 10.1038/s41588-020-0580-y

[36] O’Donnell, P. , Danahey, K. & Ratain, M. The outlier in all of us: why implementing pharmacogenomics could matter for everyone. Clin. Pharmacol. Ther. 99, 401–404 (2016). 26756170 10.1002/cpt.333 PMC4830348

[37] Wong, A.K. , Somogyi, A.A. , Rubio, J. & Philip, J. The role of pharmacogenomics in opioid prescribing. Curr. Treat. Options Oncol. 23, 1353–1369 (2022). 36001223 10.1007/s11864-022-01010-x PMC9526685

[38] Keeling, N.J. , Dunn, T.J. , Bentley, J.P. , Ramachandran, S. , Hoffman, J.M. & Rosenthal, M. Approaches to assessing the provider experience with clinical pharmacogenomic information: a scoping review. Genet. Med. 23, 1589–1603 (2021). 33927377 10.1038/s41436-021-01186-x PMC8817227

[39] Davis, B.H. & Limdi, N.A. Translational pharmacogenomics: discovery, evidence synthesis and delivery of race‐conscious medicine. Clin. Pharmacol. Ther. 110, 909–925 (2021). 34233023 10.1002/cpt.2357 PMC8662715

[40] Flanagin, A. , Frey, T. , Christiansen, S.L. & AMA Manual of Style Committee . Updated guidance on the reporting of race and ethnicity in medical and science journals. JAMA 326, 621–627 (2021). 34402850 10.1001/jama.2021.13304

[41] UF Health study to assess pharmacogenetic testing in the ER—UF Health. <https://ufhealth.org/news/2024/uf‐health‐study‐to‐assess‐pharmacogenetic‐testing‐in‐the‐er>. Accessed March 17, 2025.

[42] Cho, Y. , Jack, M. , Elahi, S. *et al*. YourPGx oncology: a novel direct‐to‐patient portal for delivering pharmacogenomic (PGx) results [conference abstract]. Clin. Pharmacol. Ther. 117(S1), S5–S123 (2025).

