# Supplementary material for: Results of the ACCOuNT Trial: A Multi‐Institutional Prospective Pharmacogenomics Implementation Trial for African American Inpatients
Source: Clin Pharmacol Ther. 2025 Aug 22;119(1):109–19. doi: 10.1002/cpt.70024 (PMC12746525; doi:10.1002/cpt.70024)
Supplement: Supplementary file 1 — Table S1 [file CPT-119-109-s001.docx]

Supplemental Table 1. Discordant Hospitalization Prescriptions

| Drug | Discordant Hospitalization Prescriptions, n (%)* |
| --- | --- |
| Pantoprazole | 67 (62.0%) |
| Carvedilol | 7 (6.48%) |
| Hydralazine | 7 (6.48%) |
| Amlodipine | 3 (2.78%) |
| Montelukast | 3 (2.78%) |
| Oxycodone | 3 (2.78%) |
| Tramadol | 3 (2.78%) |
| Aspirin | 2 (1.85%) |
| Clopidogrel | 2 (1.85%) |
| Duloxetine | 2 (1.85%) |
| Hydrochlorothiazide | 2 (1.85%) |
| Isosorbide Dinitrate | 2 (1.85%) |
| Morphine | 2 (1.85%) |
| Sildenafil | 1 (0.93%) |
| Simvastatin | 1 (0.93%) |
| Triamcinolone | 1 (0.93%) |
| Total | **108 (100%)**¶ |

* All discordant hospitalization prescriptions were categorized as genomically cautionary (yellow) prescriptions. There were no genomically contraindicated (red) hospitalization prescriptions.

¶ Across 98 admissions with discordant hospitalization prescribing.
